# Pharmacokinetics and analgesic efficacy of fentanyl and buprenorphine in chicken embryos

**DOI:** 10.1371/journal.pone.0340576

**Published:** 2026-01-08

**Authors:** Luis Truhlar, Julia Werner, Marie-Louise Schmid, Aylina Glasenapp, Heike Baehre, Judith Reiser, Julia Koerner, Heidrun Potschka, Marion Bankstahl, Christine Baumgartner

**Affiliations:** 1 Center for Preclinical Research, TUM School of Medicine and Health, Technical University of Munich, Munich, Germany; 2 Institute for Laboratory Animal Science and Central Animal Facility, Hannover Medical School, Hannover, Germany; 3 Research Core Unit Metabolomics, Hannover Medical School, Hannover, Germany; 4 Institute of Pharmacology, Toxicology, and Pharmacy, Ludwig-Maximilians-Universität München, Munich, Germany; 5 Department of Biological Sciences and Pathobiology, Centre for Biological Sciences, Pharmacology and Toxicology, University of Veterinary Medicine Vienna, Vienna, Austria; 6 Veterinary Faculty, Ludwig-Maximilians-Universität München, Munich, Germany; University of Arizona College of Medicine, UNITED STATES OF AMERICA

## Abstract

Chicken embryos are able to process and receive noxious stimuli from embryonic day (ED) 13. In the context of animal welfare, analgesic refinement is highly recommended for painful procedures from this time onwards. In this first study, the pharmacokinetic and pharmacodynamic profiles of the opioid analgesics fentanyl (0.2 mg/kg) and buprenorphine (0.5 mg/kg) were studied in ED17 chicken embryos. Analgesic plasma concentrations were determined at different time points after administration via LC-MS/MS. In a validated model of mechanical nociception in chicken embryos, analgesic efficacy and potential side effects were determined based on mean arterial blood pressure, heart rate, beak, and body movements. Maximum mean plasma levels of 37.55 ng/ml (fentanyl) and 144.17 ng/ml (buprenorphine) were reached at 15 min with a plasma elimination half-life of around 75 or 60 min, respectively. No side effects on the cardiovascular system and no alterations in body movements, reflecting possible sedative effects, were detected. No significant reduction in mean arterial pressure after noxious stimulation of the base of the beak following opioid analgesic treatment was observed. However, medium to large effect sizes may suggest potential clinical importance. A significant reduction in wide beak opening, a nociceptive behavioral parameter, was observed 15 min after administration of fentanyl. In conclusion, both opioids may have potential as safe analgesic options for the use during potentially painful procedures in chicken embryos, although further research is needed to fully demonstrate their clinical efficacy.

## 1. Introduction

Chicken embryos find use as an in vivo model in various research areas [1], like toxicity studies [1] and cancer biology regarding tumor angiogenesis, metastasis [1–3] and xenografting [4]. Their fast development, versatile usability and low costs are the main motives for choosing chicken embryos as an animal model [1,4]. Furthermore, no regulatory approval needs to be obtained, as experimental interventions on chicken embryos are not considered animal experiments in Germany and the EU as long as they do not result in hatching [5,6]. The use of chicken embryos is even considered as a more ethical choice: it is accepted as a method to replace animal experiments in the context of the 3Rs principle (Replace, Reduce, Refine) according to Russel and Burch [7]. This outdated assumption likely persisted due to the previously limited understanding and lack of detailed characterization of the onset of nociception in chicken embryos.

Recently, our research group was able to determine the 13^th^ embryonic day of chicken embryos as the earliest embryonic stage in which nociceptive stimuli can be received and processed. A physiological electroencephalogram (EEG) was clearly detected from ED13 onwards [8], and nociceptive hemodynamic and behavioral reactions were observed from the beginning of ED15/16 [9,10]. Based on these findings, the German Animal Welfare Act was amended to prohibit the killing of chicken embryos older than ED12 in the agricultural sector [11]. However, this amendment did not take chicken embryos used in research into account. Notably, many experimental procedures that are likely to cause pain are performed on chicken embryos after ED13. A recent review found that in oncological research, 47% of the analyzed studies used embryos beyond the 15^th^ day of development [12]. Information on the use of anesthesia, analgesia and killing methods is often inadequate. In contrast to chicken embryos, legal requirements to reduce burden already exist for experiments on mammalian fetuses during the last third of their normal development before birth, and for vertebrate larvae, as soon as they are able to feed independently [5,13]. Considering the current state-of-knowledge, there is an absolute necessity to limit pain, suffering, and harm inflicted on chicken embryos during an experiment from the 13th day of incubation.

Analgesics are active substances that suppress the sensation of pain by interfering with nociceptive mechanisms [14]. Despite its current importance for animal welfare, there is a lack of knowledge on analgesics in chickens and birds in general. Many drug and dosage recommendations are based on individual case reports or on pharmacokinetic studies without evidence of clinical efficacy [15]. Due to large interspecies variability in physiology, anatomy, and pharmacokinetics, extrapolating study results between different bird species is not appropriate [16]. To date, anesthetics and analgesics have been used in chicken embryos, mainly focusing on their sedative effects. An example is the immobilization to obtain high-quality Magnetic Resonance Imaging or electrocardiogram recordings without movement artifacts [17,18]. Only a few studies focused on the analgesic potential of different drug combinations in chicken embryos. Horr et al. found that the combinations of ketamine-midazolam-morphine and ketamine-xylazine are promising for chicken embryos exposed to a thermal stimulus [19]. Infiltration with lidocaine, a local anesthetic, was effective in reducing nociceptive hemodynamic reactions of chicken embryos after application of a mechanical noxious stimulus to the base of the beak [9].

Effective analgesia must be adapted to the intensity and duration of the noxious stimulus [20]. Opioids represent a highly potent class of analgesic agents [21]. They are semi-synthetic or fully synthetic substances with morphine-like effects, which are divided into opiate agonists, partial opiate agonists, opiate agonist-antagonists, and opiate antagonists [22]. Opiate agonists like fentanyl have an agonistic effect on central nervous and peripheral opiate receptors and are often used as analgesics [22]. Partial opiate agonists like buprenorphine act by displacing agonists from their receptor binding in order to become agonists themselves [22]. They are often used in veterinary medicine for acute, but also chronic pain conditions [14]. To date, studies investigating the use of fentanyl or buprenorphine in chickens remain limited and conflicting. While some demonstrated an anesthesia-sparing effect of opioids in adult chickens [23], others have not confirmed these findings [24]. Transdermal fentanyl patches resulted in plasma fentanyl levels expected to provide analgesia in humans, but unfortunately, clinical effectiveness was not investigated [25]. Intraarticular injections of fentanyl or buprenorphine for the treatment of chicken with joint pain were ineffective in relieving pain [26]. However, since opioids are characterized by high organ tolerance and low embryotoxic potential, their use in chicken embryos is promising [22].

To ensure the welfare of chicken embryos in research, suitable analgesia protocols must be established. The aim of this study was to evaluate efficacy, cardiovascular safety and potential sedative effects of opioid-based analgesia in ED17 chicken embryos. Therefore the opioids fentanyl and buprenorphine were investigated considering pharmacokinetic and pharmacodynamic characteristics in ED17 chicken embryos.

## 2. Materials and methods

According to Directive 2010/63/EU of the European Parliament and the German Animal Welfare Act, no ethical approval was required for the use of chicken embryos in these experiments. All experiments were performed in an AAALAC-accredited facility and conducted in strict accordance with the institutional guidelines for the care and use of laboratory animals and general animal welfare principles, including the humane euthanasia of embryos.

### 2.1. Animals

Fertilized white chicken eggs *(Gallus gallus domesticus)* of the breed Leghorn White from a specified pathogen-free (SPF) livestock farm (VALO BioMedia GmbH, Osterholz-Scharmbeck, Germany) were used for the study.

The chicken eggs were delivered chilled and in a vertical position with the blunt pole facing upwards. Before the start of incubation (defined as ED0) at 8:30 a.m., the eggs were stored at room temperature for 24 hours (h). The eggs were incubated (Favorit-Olymp 192 Spezial, HEKA-Brutgeräte, Rietberg, Germany) at a temperature of 37.8°C and at a relative humidity of 55% until ED17, i.e., the day of experimental use. In addition, the eggs were turned six times a day at regular intervals until fenestration at ED3.

On the 3^rd^ day of incubation, the chicken eggs were put in a horizontal position for at least two minutes (min). 5–6 ml of albumin were extracted by piercing the eggshell at the pointed end with a 5-ml syringe (Injekt® Luer Solo 5 ml, B. Braun Melsungen AG, Melsungen, Germany) with an attached 18 G cannula (Sterican® 18 G, B.Braun Melsungen AG, Melsungen, Germany). To ensure the necessary stability for the following steps, a sufficiently large piece of adhesive tape (Leukosilk® S, BSN medical GmbH, Hamburg, Germany) was applied to the horizontal top of the egg. Then, a 2 x 2 cm piece of eggshell was cut out with scissors. 0.5 ml Penicillin-Streptomycin (Penicillin-Streptomycin, P4333-100ML, Sigma-Aldrich, Sigma-Aldrich Chemie GmbH, Taufkirchen, Germany) were dripped on the chorioallantoic membrane (CAM) after visual determination of embryonic vitality. The eggshell was sealed with cling film (Saran™ Cling Plus® Wrap, S.C. Johnson & Son, Inc., WI, USA). Further incubation took place in a horizontal position until ED17.

The chicken embryos were visually checked daily for vitality until conduct of the experiment. At the end of the experiment, the chicken embryos were euthanized by intravenous (i.v.) or intraperitoneal (i.p.) injection of 25 mg Sodium Pentobarbital (Euthadorm® 500 mg/ml, CP-Pharma Handelsgesellschaft mbH, Burgdorf, Germany). Chicken embryos were additionally decapitated to ensure their death, after which their weight and sex were determined including macroscopical assessment of the presence of gonads.

### 2.2. Experimental design

A priori power analysis was carried out ahead of the pharmacodynamics study. Assuming a difference of 9% in the mean arterial blood pressure between the analgesia group and the control group, with a two-sided significance of 0.01 and a power of 0.8, a group size of n = 10 per group was required. To ensure proper randomization, we used n = 13 embryos for the control groups. In the pharmacodynamics study, the experimenter was blinded and eggs were randomly assigned to the different study groups using a software program. All experiments were performed under standardized conditions in a custom-made heat chamber equipped with a red light lamp (ARTAS GmbH, Arnstadt, Germany). The eggs were placed on metal beads (Lab Armor Beads, Sheldon Manufacturing, Cornelius, NC, USA) heated up to 38–40°C in a silicone bowl, which was placed on a heating mat (ThermoLux, Witte + Sutor GmbH, Murrhardt, Germany) for additional warming. To regulate the relative humidity, water was regularly nebulized. In order to limit the developmental deviation of the chicken embryos, all experiments took place between 8:30 a.m. and 5 p.m.

### 2.3. Analgesics

Embryonated eggs (ED17) were weighed in order to calculate the application volume for fentanyl and buprenorphine. 0.2 mg/kg egg weight fentanyl (Fentadon® 50 µg/ml, Dechra Veterinary Products Deutschland GmbH, Aulendorf, Germany) or 0.5 mg/kg egg weight buprenorphine (Bupresol® vet. Multidose 0.3 mg/ml, CP-Pharma Handelsgesellschaft mbH, Burgdorf, Germany) were applied to the air chamber. In preliminary investigations (S1 Fig), fentanyl was also administered via the CAM. For the pharmacodynamic study, a control group received an administration of saline as the corresponding vehicle solution at each pre-treatment time point (Isotonische Natriumchlorid-Lösung ad us. vet. B. Braun Vet Care, B. Braun Melsungen AG, Melsungen, Germany). These control groups are referred to as NaCl groups throughout the manuscript.

### 2.4. Pharmacokinetics

Plasma concentrations of fentanyl and buprenorphine in chicken embryos were measured at different time points after application into the air chamber. Blood sampling was performed under a dissecting microscope (Stemi SV6, Zeiss, Oberkochen, Germany) using custom-made catheters, consisting of a 0.5 ml insulin syringe (0,5 ml BD Micro-Fine™ + , Becton, Dickinson and Company, Franklin Lakes, USA), a 13–15 cm long piece of rubber hose (30m Portex® Non Sterile Polythene Tubing, 0.28 mm ID, 0.61 mm OD, Smiths Medical International Ltd., Hythe, Kent, UK) with an attached needle (Sterican® 30G, B.Braun Melsungen AG, Melsungen, Germany). For fentanyl, blood samples were collected from n = 25 eggs at the time points 5 min (n = 10 blood samples), 15 min (n = 11), 30 min (n = 8), 45 min (n = 3), 60 min (n = 4) and 120 min (n = 4) post injectionem (p.i.). Due to the small number of blood samples at 45 min (n = 3) resulting (probably by chance) in data points that were all in the lower variance range these data were excluded from pharmacokinetic and correlation analyses. For buprenorphine, blood sampling was carried out 15 min (n = 7), 30 min (n = 7), 60 min (n = 7) and 120 min (n = 7) p.i., which required a number of n = 19 eggs. One to three blood samples were taken from each egg. A minimum volume of 70 µl per sample was required for the analytical procedure. For this part of the study, a total number of n = 44 eggs were used.

Blood samples were transferred to a 200 µl EDTA-tube (Microvette® 200 K3E, SARSTEDT AG & Co. KG, Nümbrecht, Germany) and centrifuged (Centrifuge 5415 R, Eppendorf SE, Hamburg, Germany) for 10 min at 2500 g and 4°C. Supernatant plasma was collected and a sample and a backup-sample, each 25 µl, were pipetted into safe-seal tubes (SafeSeal tube 1,5 ml, SARSTED AG & Co. KG, Nümbrecht, Germany). Plasma samples were stored at −80°C until shipping on dry ice to the Institute for Laboratory Animal Sciences and Central Animal Facility in Hannover, where further processing and measurement of the plasma concentrations took place.

### 2.5. Sample preparation for liquid chromatography mass spectrometry analysis

The plasma concentrations of fentanyl and buprenorphine were determined using liquid chromatography coupled with mass spectrometry (LC-MS/MS). The LC system used, made by Shimadzu (Duisburg, Germany), consisted of LC-30AD HPLC pumps, a SIL-30 AC temperature-controlled autosampler, a DGU-20A5 degasser, a CTO-20 AC oven, and a CBM-20A control unit. The analgesics were detected using triple quad mass spectrometry (5500QTRAP; Sciex, Framingham, Massachusetts). For the extraction of fentanyl and buprenorphine, 25 µl of chicken plasma was added to 100 µl of ice-cold extraction solution (acetonitrile/methanol 1/1, v/v). This contained 6.25 nM of the internal standard moclobemide. After storage overnight at −20°C, the samples were centrifuged for 10 min at 20,800 x g and at 4°C. The supernatant was then transferred to 2 ml reaction tubes and evaporated under nitrogen at 40°C to dryness. The analytes and the internal standard were then redissolved in 100 µl methanol/water (1/1, v/v). After further centrifugation (10 min, 20,800 x g, 4°C), the samples were placed in analysis vials with inserts and analyzed by mass spectrometry. For later quantification of fentanyl and buprenorphine, calibrator series were prepared in chicken plasma. The calibrator series consisted of 12 points and covered a concentration range of 0.49–1000 nM. The calibrator series was prepared in an identical way to the samples.

### 2.6. LC-MS/MS analysis

Ten µl of the prepared sample were injected into the LC-MS/MS system. Separation was performed using reversed phase chromatography on a C-18 column (ZORBAX Eclipse XDB-C18 1.8 µ, 50 x 4.6 mm, Agilent, Santa Clara, California, USA) preceded by a C18 Security Guard (Phenomenex, Aschaffenburg, Germany). The column temperature was maintained at 40°C throughout the analysis. Water (solvent A) and methanol (solvent B) were used as solvents. Both contained 0.1% formic acid. At the start of elution, the proportion of solvent B was set to 20%. Within 7 min, the proportion was increased linearly to 95%. This composition was maintained for 3 min before the column was re-equilibrated for another 3 min. The total analysis time was therefore 13 min at a constant flow rate of 0.4 ml/min. Under these conditions, the retention time of fentanyl was 5.5 min, that of buprenorphine 5.3 min, and that of moclobemide 3.6 min. The analytes were detected by mass spectrometry in positive ionization mode (ESI) at 650°C. Fentanyl was quantified using the mass transition 337.4 to 188, and buprenorphine using the mass transition 468.4 to 396.1. For the internal standard moclobemide, the mass transition 269–182 was used. Control of HPLC and the mass spectrometer as well as data sampling was performed by Analyst software (version 1.7., Sciex). For quantification, calibration curves were created by plotting peak area ratios of both analytes and the internal standard versus the nominal concentration of the calibrators. The calibration curve was calculated using quadratic regression and 1/x weighing. The lower limit of quantification was 0.2 ng/ml for fentanyl and 1.15 ng/ml for buprenorphine, respectively.

### 2.7. Pharmacokinetics analysis

Pharmacokinetic analysis was performed using the PKanalix application (Version 2024R1, Lixoft, Antony, France). Noncompartmental analysis was applied for determination of plasma elimination half-lives of fentanyl and buprenorphine and other key pharmacokinetic parameters. The mode for sparse data was applied and the following pharmacokinetic parameters estimated: maximum plasma concentration (C_max_), time of maximum plasma concentration (T_max_), plasma elimination half-life (T_1/2_), area under the curve from time 0 extrapolated to infinity (AUC 0→∞), area under the first moment curve from time 0 extrapolated to infinity (AUMC 0→∞), mean residence time from time 0 extrapolated to infinity (MRT 0→∞), volume of distribution (Vd/F), and clearance (CL/F).

### 2.8. Pharmacodynamics

Clinical analgesic efficacy of fentanyl and buprenorphine in chicken embryos at ED17 was evaluated using hemodynamic parameters. In addition, the effect of the analgesics on behavioral parameters was examined. N = 10 animals were examined per time point (15, 30, 60 min) and per analgesic (fentanyl, buprenorphine), n = 13 animals were investigated per time point (15, 30, 60 min) for the control group (NaCl). For the installation of the measuring catheters, a CAM artery was identified, and a Desmarres lid retractor, clamped into a mobile holding device, was placed underneath the vessel. A microtip-catheter (FISO-LS Fiber Optic Pressure Sensor, FOP-LS-PT9–10, FISO Technologies Inc., Quebec, QC, Canada) was calibrated to zero pressure using the Evolution software, with the sensor immersed in water at the level of the embryo´s vasculature. Then the CAM artery was cut open with fine scissors (FST 15000−03, Fine Science Tools Inc., Foster City, CA, USA) and the microtip-catheter to measure mean arterial pressure (MAP) and heart rate (HR) was inserted. The beak of the chicken embryo was placed on a swab (Raucotupf, Lohmann & Rauscher International GmbH & Co. KG, Rengsdorf, Germany) to make it accessible for the noxious stimulation of the beak.

At defined time points after the administration of the analgesic or NaCl control a noxious, mechanical stimulus (Pinch) and a non-noxious control stimulus (Touch) were applied to the base of the beak of the chicken embryo. Systolic arterial pressure (SAP), diastolic arterial pressure (DAP), MAP and HR were measured and recorded every four seconds (sec) (PLUGSYS module, EIM-B, EIM-A, HAEMODYN Software v 2.0, Hugo Sachs Elektronik – Harvard Apparatus GmbH, March-Hugstetten, Germany, Evolution Software v 2.1.6.0, FISO Technologies Inc., Quebec, QC, Canada). To record the behavioral parameters, the number of each embryo`s body movements (consisting of embryonic movement of beak, head, body, wings) and wide beak openings were observed visually and counted manually. A previous study found the wide beak opening to be a recurring reaction to the Pinch [10]. It is characterized by a separate, wide opening of the upper and lower sides of the beak with the moving tongue becoming visible [10]. For the Pinch, an Analgesia Meter (BIO-PR-M, Bioseb, Vitrolles, France) was attached at the base of the beak and compressed. The device, which recorded the applied pressure, was adjusted by attaching the tips of a mosquito clamp. For the Touch, the base of the beak was lightly touched by the Analgesia Meter with the clamps closed. The stimuli were always performed by the same person to ensure a high degree of standardization.

Before the Pinch, a resting phase of 3.5 min, followed by a 1 min baseline (BL) and 30 sec of preparation time for the stimulus were measured. After the Pinch, 3.5 min were measured, followed by 1 min BL and 30 sec preparation time prior to the Touch, after which another 2.5 min were recorded.

To rule out reduced responsiveness to the stimulus due to too low temperature, the temperature inside the egg was measured before the resting phase and after the trial was finished.

### 2.9. Statistical analysis

#### 2.9.1. Pharmacodynamics analysis.

SAP, DAP, MAP and HR were recorded every four sec using HAEMODYN software. After converting the raw data to Excel (Microsoft® Excel® 2016 MSO (16.0.4266.1001) 64-Bit), the means were calculated for one min before the mechanical stimulus (BL) and one min after the stimulus (Pinch/Touch). After that, the percentage deviation of the MAP and HR after stimulus (Pinch/Touch) to the previous BL were calculated. Absolute values were used, as each deviation from the BL was evaluated as a reaction to the stimulus. Individual values had to be excluded due to measurement errors, especially after the Touch. To check for statistical significance, a statistical analysis was carried out using the software GraphPad Prism 10.3.0 (507). The normal distributions of the data were tested by the Shapiro-Wilk test. For multiple group comparisons, normally distributed data were analyzed by a one-way ANOVA test, for not normally distributed data a Kruskal-Wallis test was performed. Data that showed significance were further tested using the post-hoc Tukey test (for ANOVA) and the post-hoc Dunn test (for Kruskal-Wallis).

The number of body movements and wide beak openings was counted over one min before the mechanical stimulus (BL) and one min after the mechanical stimulus (Pinch). Within each test group, the means of all counted movements were calculated. The number of embryos of the different groups that showed wide beak opening was statistically evaluated using the software IBM SPSS Statistics 30.0.0.0 (171). Data that reached significance in in the Fisher-Freeman-Halton test were further analyzed using a post-hoc Fisher test with Bonferroni correction for multiple testing.

## 3. Results

### 3.1. Group homogeneity

Mean egg weights ± standard deviation (SD) of the pharmacokinetic subgroups per time point ranged from 41.70 ± 5.18 to 46.90 ± 0.92 grams (g) for fentanyl and from 49.80 ± 3.27 to 53.77 ± 1.96 g for buprenorphine. The embryo weights (mean ± SD) ranged from 14.33 ± 2.58 to 17.47 ± 0.76 g for fentanyl and from 14.57 ± 4.57 to 15.07 ± 4.03 g for buprenorphine. No significant differences were found for egg and embryo weight within fentanyl or buprenorphine groups. The sex distribution varied within the groups. An overview is presented in the Supplements (S1 Table).

Mean egg weights ± SD of the pharmacodynamic groups ranged from 41.97 ± 2.04 to 48.29 ± 4.56 g (S2 Table). Significant differences in egg weight were observed between Fentanyl60 and NaCl15 (p = 0.0016), Buprenorphine15 (p = 0.0293), NaCl30 (p = 0.0222) as well as Buprenorphine30 (p = 0.0094). Embryo weights did not differ between the groups. An overview is shown in the Appendix (S2 Table). The mean pinching pressure ± SD applied across all groups (NaCl, fentanyl, buprenorphine) was 1263 ± 267.6 g with no significant differences among the groups.

### 3.2. Pharmacokinetics of fentanyl and buprenorphine in chicken embryos

Prior to the pharmacokinetic study, the administration route for analgesics was pre-tested in a small number of embryos (n = 18). Therefore, 0.2 mg/kg fentanyl per egg weight was applied via the air chamber (n = 8) or the CAM (n = 10), and plasma concentrations were determined using LC-MS/MS at 5, 15, 60, and 120 min after administration (S1 Fig). Both administration routes resulted in quantifiable plasma concentrations at all investigated time points. In the following experiments, analgesics were administered via the air chamber.

**Fig 1:**
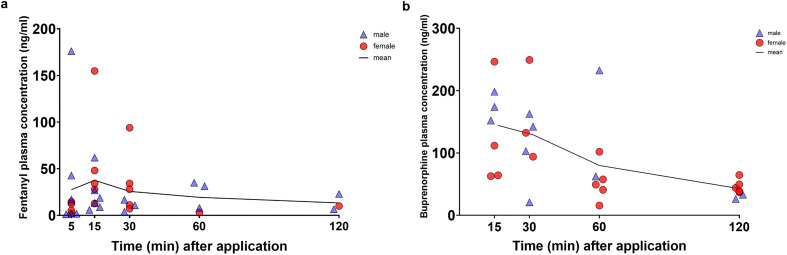
Plasma concentration of fentanyl and buprenorphine after administration via air chamber. a Fentanyl plasma concentrations (ng/ml) at 5 (n = 10), 15 (n = 11), 30 (n = 8), 60 (n = 4), and 120 (n = 4) min after application of 0.2 mg/kg (egg weight) via air chamber are presented as individual data points and mean (line). b Buprenorphine plasma concentrations (ng/ml) at 15, 30, 60, and 120 min (n = 7 per time point) after application of 0.5 mg/kg (egg weight) via air chamber are presented as individual data points and mean (line).

Plasma concentrations were determined at 5, 15, 30, 60, and 120 min after administration of 0.2 mg/kg fentanyl per egg weight (Fig 1a). Mean administered dose per embryo weight was 0.58 ± 0.08 mg/kg, ranging between 0.45 and 0.86 mg/kg. The highest average plasma levels were measured at 15 min with 37.55 ± 42.51 ng/ml. A high range of plasma concentrations was observed at all investigated time points, reflected by a minimum of 1.1 ng/ml to a maximum of 176.0 ng/ml fentanyl at 5 min. Pharmacokinetic parameters are provided in Table 1.

**Table 1:** Noncompartmental analysis of pharmacokinetic parameters after administration of fentanyl or buprenorphine via air chamber. C_max_, maximum plasma concentration; T_max_, time of maximum plasma concentration; T_1/2_, plasma elimination half-life; AUC 0→∞, area under the curve from time 0 extrapolated to infinity; AUMC 0→∞, area under the first moment curve from time 0 extrapolated to infinity; MRT 0→∞, mean residence time from time 0 extrapolated to infinity; Vd/F, volume of distribution; CL/F, clearance.

Parameter	Unit	Mean Fentanyl	Mean Buprenorphine
C_max_	ng/ml	37.55	144.17
T_max_	min	15.00	15.00
T_1/2_	min	76.40	57.28
AUC 0→∞	min/ng/ml	3,990.43	1,3373.00
AUMC 0→∞	min²/ng/ml	3,463,097.65	1,185,689.08
MRT 0→∞	min	116.05	88.66
Vd/F	ml/kg	16,319.92	3,089.85
CL/F	ml/min/kg	148.06	37.39

After administration of 0.5 mg/kg buprenorphine, plasma concentrations were determined at 15, 30, 60, and 120 min (Fig 1b). Mean administered dose per embryo weight was 1.84 ± 0.47 mg/kg, ranging between 1.17–3.12 mg/kg. The highest average plasma levels were measured at 15 min with 144.2 ± 68.86 ng/ml. Also, for buprenorphine, a high range of plasma concentrations was observed at all investigated time points, ranging between a minimum of 15.57 ng/ml at 60 min to a maximum of 249.26 ng/ml at 30 min after administration. Pharmacokinetic parameters are provided in Table 1.

**Fig 2:**
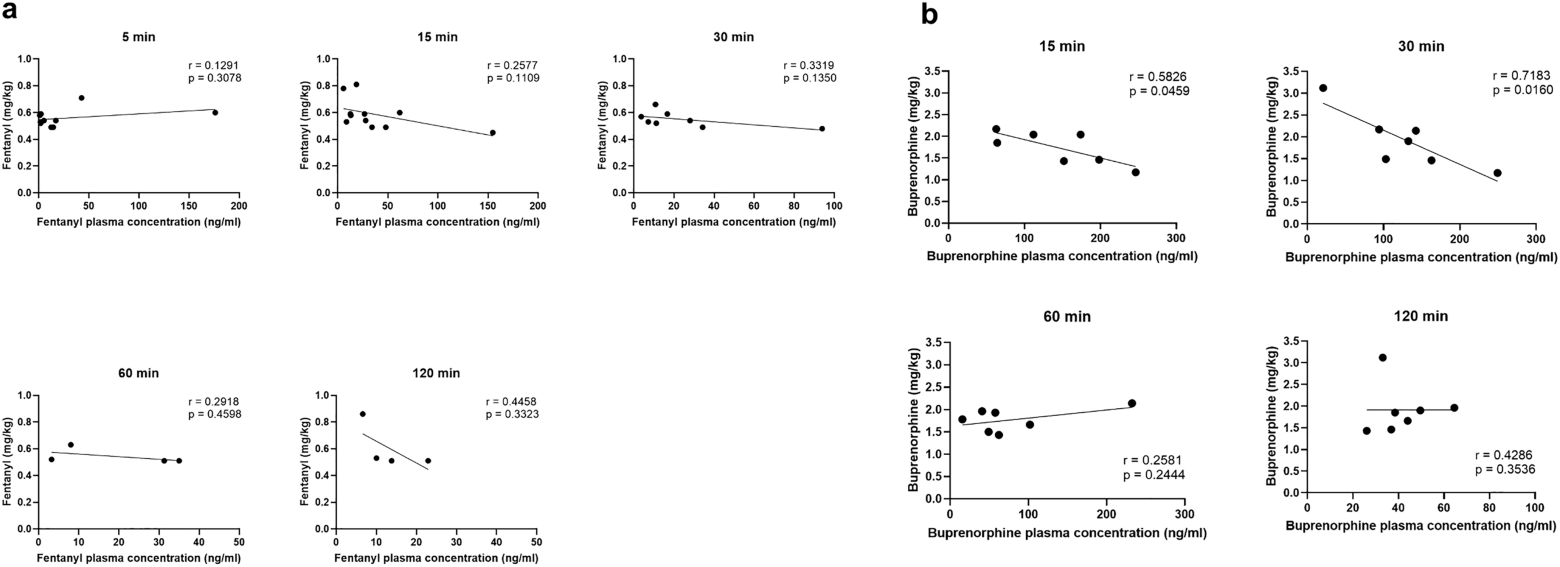
Correlation analysis of individual fentanyl und buprenorphine plasma concentrations and the dose administered per embryo at the time points examined after application via the air chamber. a Pearson correlation analysis of fentanyl plasma concentration (ng/ml) at 5 (n = 10), 15 (n = 11), 30 (n = 8), 60 (n = 4), and 120 (n = 4) min after application of 0.2 mg/kg (egg weight) via the air chamber and individual calculated dose per embryo (mg/kg) is presented as individual data points and simple linear regression (line). b Pearson correlation analysis of buprenorphine plasma concentrations (ng/ml) at 15, 30, 60, and 120 min (n = 7 per time point) after application of 0.5 mg/kg (egg weight) via air chamber and individual calculated dose per embryo (mg/kg) is presented as individual data points and simple linear regression (line).

The applied dose of analgesics was based on egg weight. However, a variation in embryo weight and therefore in applied dose per embryo weight was observed. Correlation analysis of plasma concentration and applied dose per embryo weight for each time point and drug was performed (Fig 2). For fentanyl, no significant correlation was observed (Fig 2a). For buprenorphine, correlation analysis revealed a significant relation at 15 (r = 0.5826, p = 0.0459) and 30 (r = 0.7183, p = 0.0160) min after administration (Fig 2b).

### 3.3. Effect of fentanyl on the MAP response to a noxious stimulus

**Fig 3:**
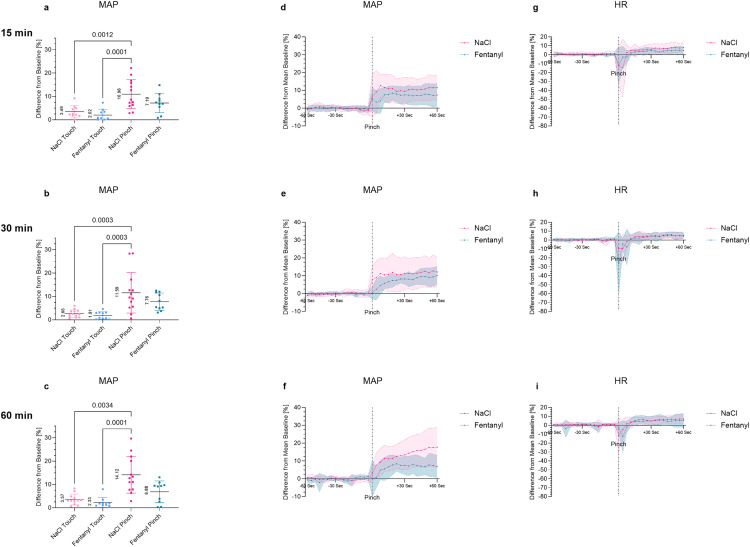
Percent change in MAP and HR after mechanical stimulation with fentanyl. ED17 embryos received a mechanical stimulus (Pinch/Touch) at different time points (15, 30, 60 min) after administration of NaCl or fentanyl. **(a-c)** Percent change from BL in MAP after Pinch and Touch. Values are shown as the mean ± SD. **(d-f)** Percent change in MAP over time. Values one min before and one min after Pinch are shown as the mean ± SD (shaded). **(g-i)** Percent change in HR over time. Values one min before and one min after Pinch are shown as the mean ± SD (shaded). Normally distributed data were analyzed by ordinary one-way ANOVA **(a, b)**, non-normally distributed data by a Kruskal-Wallis test **(c)**. A p-value of <0.01 was considered statistically significant.

Responses of MAP and HR to a stimulus (Pinch/Touch) were measured 15, 30 and 60 min after the administration of NaCl or fentanyl. The groups that received NaCl, as a control, showed the largest increases after Pinch (Fig 3a-f). At all three time points after NaCl application, the MAP significantly increased after Pinch compared to the Touch controls (Fig 3a-c). After the Touch, only small deviations of MAP, around 3% (NaCl) and 2% (fentanyl) in mean, were observed, which correspond to physiological fluctuations in blood pressure (BP). The administration of fentanyl 15, 30 or 60 min before stimulation resulted in an increase in MAP after Pinch to below 10% (Pinch 15 min: NaCl 10.96 ± 6.24, Fentanyl 7.19 ± 4.13, Pinch 30 min: NaCl 11.59 ± 8.68, Fentanyl 7.76 ± 3.68, Pinch 60 min: NaCl 14.12 ± 7.83, Fentanyl 6.88 ± 4.62) (Fig 3a-f) but did not lead to a statistically significant reduction in MAP compared to the NaCl groups. It must be pointed out that high variance with standard deviations of over ± 5 was observed particularly in the NaCl controls. However, medium to large effect sizes were observed (g = 0.69 at 15 min, g = 0.55 at 30 min and g = 1.09 at 60 min). An overview of the calculated Hedges` g effect sizes of MAP and HR is presented in the appendix (S3 Table a, c).

A drop in HR shortly after the Pinch and a slight increase in HR in the further course of the measurements were observed in all fentanyl treated groups and NaCl controls at all time points (Fig 3g-i). Observed differences in HR changes between the groups can be found in the Supporting Information (S2 Fig a,c,e).

### 3.4. Effect of buprenorphine on the MAP response to a noxious stimulus

**Fig 4:**
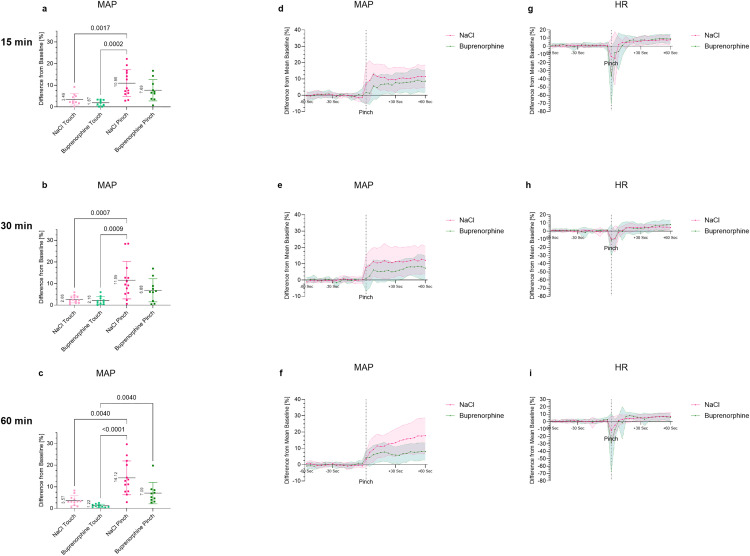
Percent change in MAP and HR after mechanical stimulus with buprenorphine. ED17 embryos received a mechanical stimulus (Pinch/Touch) at different time points (15, 30, 60 min) after administration of NaCl or buprenorphine. **(a-c)** Percent change in MAP after Pinch and Touch. Values are shown as the mean ± SD. **(d-f)** Percent change in MAP over time. **(g-i)** Percent change in HR over time. Values one min before and one min after Pinch are shown as the mean ± SD (shaded).

The response of MAP and HR to a noxious stimulus after administration of buprenorphine was compared to NaCl and Touch controls. At all time points, the NaCl Pinch group showed the greatest deviation in MAP as a response to the noxious stimulus (Fig 4). At 15, 30 and 60 min post drug administration, the MAP of NaCl Pinch was significantly increased compared to NaCl Touch and Buprenorphine Touch (Fig 4a-c). In addition, the Buprenorphine Pinch group showed a significant increase in MAP compared to Buprenorphine Touch at 60 min. Buprenorphine did not result in a statistically significant reduction in MAP compared to the NaCl control (Pinch 15 min: NaCl 10.96 ± 6.24, Buprenorphine 7.69 ± 4.94 (g = 0.57), Pinch 30 min: NaCl 11.59 ± 8.68, Buprenorphine 6.88 ± 5.35 (g = 0.63), Pinch 60 min: NaCl 14.12 ± 7.83, Buprenorphine 7.09 ± 4.95 (g = 1.04). As mentioned, high variance was noticeable, especially in the NaCl groups. The appendix contains an overview of the effect sizes for MAP and HR calculated according to Hedges’ g (S3 Table b, d). In all groups, HR decreased directly after Pinch and then showed a slight increase over time. An overview of the percent change in HR is shown in the Supporting Information (S2 Fig b, d, f).

Normally distributed data were analyzed by ordinary one-way ANOVA (a, b), non-normally distributed data by a Kruskal-Wallis test (c). A p-value of <0.01 was considered statistically significant.

### 3.5. Fentanyl and buprenorphine show no depressive effect on cardiovascular parameters

In order to detect cardiovascular side effects of the applied dosages of fentanyl and buprenorphine, mean HR and BP were compared at 15, 30, and 60 min after NaCl or opioid application. Application of fentanyl or buprenorphine showed no effect on BP and HR compared to NaCl controls (Table 2). Significant differences in HR were only observed between Fentanyl 15 min and Fentanyl 30 min (p = 0.0299).

**Table 2:** Effects of fentanyl and buprenorphine on cardiovascular parameters. Systolic (SAP), diastolic (DAP), mean arterial pressure (MAP), and heart rate (HR) of chicken embryos on ED17 at 15, 30, and 60 min after application of NaCl, fentanyl, or buprenorphine. Values are shown as the mean ± SD. Normally distributed data (HR, MAP, DAP) were analyzed using ordinary one-way ANOVA. For nonparametric data (SAP) a Kruskal-Wallis test was performed. Superscripts within the HR row indicate significant differences between groups (p < 0.05). Significant differences in HR were observed between Fentanyl 15 min and Fentanyl 30 min.

	15 min			30 min			60 min		
	NaCl	Fentanyl	Buprenorphine	NaCl	Fentanyl	Buprenorphine	NaCl	Fentanyl	Buprenorphine
SAP (mmHg)	19.40 ± 5.07	18.98 ± 4.79	21.29 ± 5.09	20.32 ± 2.19	21.17 ± 3.19	22.01 ± 5.42	18.75 ± 2.83	19.45 ± 5.11	21.55 ± 3.71
DAP (mmHg)	8.27 ± 3.75	8.11 ± 2.76	10.54 ± 4.99	8.82 ± 2.58	9.92 ± 2.04	10.61 ± 4.74	7.66 ± 2.32	8.49 ± 2.89	9.13 ± 2.03
MAP (mmHg)	13.35 ± 4.19	12.91 ± 3.61	15.48 ± 4.83	13.97 ± 2.21	15.19 ± 2.37	15.77 ± 4.83	12.68 ± 2.30	13.43 ± 3.51	14.85 ± 2.75
HR (bpm)	177.84 ± 27.34	161.86* ± 19.42	188.16 ± 35.15	168.84 ± 26.08	197.85* ± 15.47	170.65 ± 22.04	181.91 ± 20.11	170.03 ± 26.59	177.88 ± 15.73

### 3.6. Effects of fentanyl and buprenorphine on behavioral parameters

**Fig 5:**
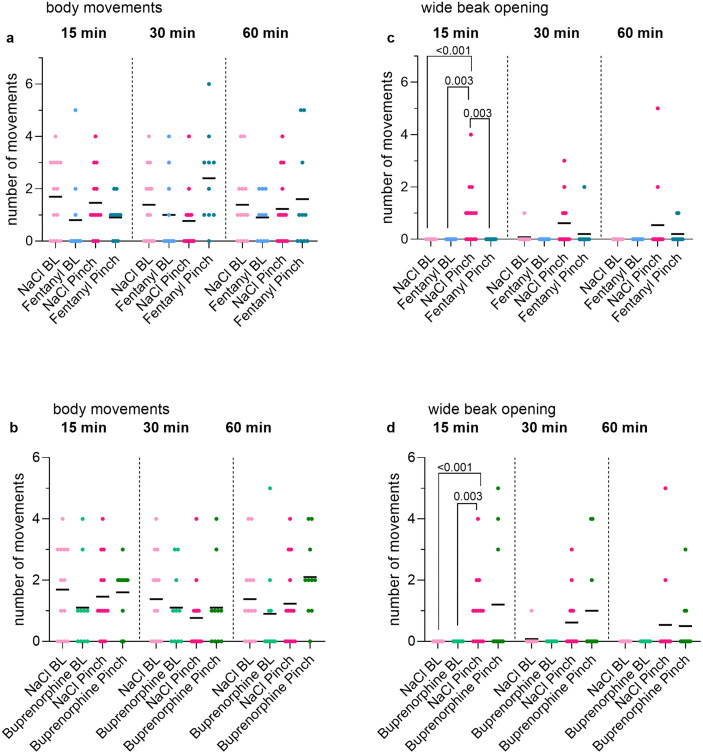
Effects of fentanyl and buprenorphine on behavioral parameters. Number of body movements (a, b) and wide beak openings (c, d) of ED17 chicken embryos at 15, 30 and 60 min after injection of NaCl **(a-d)**, fentanyl (a, c) and buprenorphine (b, d) during one min before (BL) and one min after Pinch. The absolute number of body movements and wide beak openings per embryo and their calculated means are shown. **(c, d)** Significances shown result from the comparison of the total number of embryos per group that showed wide beak opening. Data that reached significance in the Fisher-Freeman-Halton test were further analyzed using a post-hoc Fisher test with Bonferroni correction for multiple testing.

Body movements of the chicken embryos were observed to monitor potential sedative effects of the analgesics. In addition, the number of wide beak opening was analyzed as a nocifensive parameter. With regard to the body movements, activity was observed independently of the substance administered (NaCl, fentanyl, buprenorphine), the time point (15, 30, 60 min) and the measurement phase (BL, Pinch) (Fig 5a, b). During BL, only one animal of the NaCl group at 30 min showed a single wide beak opening (Fig 5c, d). After noxious stimulation the number of wide beak openings increased equally in the NaCl Pinch and Buprenorphine Pinch group (Fig 5d). After Pinch at 15 min the number of embryos that showed wide beak opening increased significantly in the NaCl group compared to NaCl BL (p < 0.001) and to Buprenorphine BL (p = 0.003) (Fig 5d). Similar observations were also made for NaCl BL vs NaCl Pinch (p < 0.001), Fentanyl BL vs NaCl Pinch (p = 0.003) and also for Fentanyl Pinch vs NaCl Pinch (p = 0.003) (Fig 5c).

## 4. Discussion

Opioid analgesics can provide strong analgesia for the treatment of acute and chronic pain conditions in veterinary medicine, in particular in mammals [20,27]. They may also have potential in avian medicine, but a lack of reliable pharmacokinetic data and studies on clinical efficacy using standardized nociceptive stimuli leads to uncertainties regarding their use [20,28].

Given the limited research on opioid use in chickens, the effective dosages and plasma concentrations required for an anti-nociceptive effect remain unknown. Although empirical data are scarce, commonly cited dosages in avian medicine are 0.01–0.02 mg/kg for fentanyl and 0.01–0.05 mg/kg for buprenorphine [29]. Taking into account the principle of allometric scaling, small birds often need a comparatively higher dose than larger ones [29]. Moreover, previous reports indicate that juvenile animals require higher dosages than adult animals to achieve comparable effects [30]. To comply with this consideration, we applied a 10-fold higher dosage for both drugs, resulting in 0.2 mg/kg fentanyl and 0.5 mg/kg buprenorphine. After conducting a pilot study on the different administration routes, the air chamber was selected as the route of application for the main study. The successful and complete injection of a substance into the air chamber of chicken eggs on ED6 - ED18 was demonstrated without exception in an earlier study [31]. In comparison, the application onto the CAM and into blood vessels showed age-dependent success rates [31]. In addition to dose selection, this decision also reflected the need to minimize the injection volume. Since the air chamber can accommodate only a limited amount of fluid, it was essential to prevent overflow into the CAM, which might have altered pharmacokinetic data by enabling unintended absorption through the CAM. Inoculation into the air chamber was also preferable due to the specific requirements of the pharmacodynamic study design. To allow stimulation at the base of the beak, it was necessary to open the embryonic membranes, including the CAM. This intervention might have disrupted local tissue integrity and might have interfered with drug distribution if administration had occurred via CAM or blood vessels.

The eggshell membrane is a microporous mesh consisting of fibers with macropores sized up to 5 µm [32]. It appears to be more permeable to hydrophilic than to lipophilic substances [33]. Since fentanyl and buprenorphine are very lipophilic [14], commercially available preparations with fentanyl citrate and buprenorphine hydrochloride, which were used in our study, are established for faster absorption and onset of effect [34,35]. We were able to confirm rapid resorption of the opioids from the air chamber, as the highest blood plasma concentrations were measured for both analgesics at 15 min after application to the air chamber. A rather variable drug resorption with a broad range of individual plasma concentration was detected, ranging between 1.1 ng/ml to 176.0 ng/ml (both at 5 min) for fentanyl and between 15.57 ng/ml (at 60 min) to maximum 249.26 ng/ml (at 30 min) for buprenorphine. While a significant correlation between plasma concentration and applied dose per embryo was found at 15 and 30 min for buprenorphine, none was observed for fentanyl. The variance in plasma concentrations can be explained by the rather high drug dose range applied, i.e., 0.45 to 0.86 mg/kg for fentanyl and 1.17 to 3.12 mg/kg for buprenorphine. The administration volumes of drug solutions were calculated based on egg weight as it is only possible to determine the actual embryo bodyweight when the experiments are finished, resulting in the respective variance. Another contributing factor may be the chosen administration route, as the compounds were not administered directly into a blood vessel but via the air chamber. The plasma concentration variability and lack of proportional increase of plasma concentrations with the dose administered (for fentanyl and buprenorphine, 60 and 120 min) may also be explained by the rapid development of the chicken embryos. For this reason, the standardized data collection took place within a period of a few hours per day, which could, however, already lead to such differences. Also, sex and inter-individual differences of the embryos in metabolism, distribution and elimination of the drugs might play a role. Delaski et al. observed high individual variability in fentanyl plasma concentrations following transdermal application in adult chickens [25]. These differences in fentanyl absorption compared to mammals have been linked to the high body temperature of birds [25]. Although minimizing the risk of external influences through standardized incubation parameters, individual embryo temperatures were not measured and therefore differences cannot be ruled out as a possible cause for the variable plasma concentrations. The pharmacokinetic part of this study is of exploratory nature and aims to provide initial pharmacokinetic parameter estimates to inform follow-up studies. In view of the interindividual variance of the opioid plasma levels a larger n seems preferable in future studies.

Fentanyl plasma concentrations from 0.2–1.2 ng/ml are considered therapeutically effective in humans [25,36]. In adult chickens, similar plasma levels of fentanyl were achieved by transdermal application within 2–4 h [25]. Compared to this, we achieved significantly higher plasma levels in the chicken embryos in our study. The highest fentanyl plasma concentrations were measured at 15 min with 37.55 ± 42.51 ng/ml. Analgesia by buprenorphine has been described in humans at plasma levels of 1 ng/ml and below [37]. Those plasma concentrations could be reached or even exceeded in some bird species and are considered effective in relieving pain at >1 ng/ml [16]. Buprenorphine plasma concentrations were measured in brain, lung, liver, blood and yolk after injection into the chorioallantoic sac in chicken embryos on ED13 [38]. Interestingly, the highest blood plasma concentrations were observed 20 min after application, closely aligning with our finding of a C_max_ of 144.17 ng/ml after 15 min [38].

It is important to note that measurable plasma concentrations alone do not constitute evidence of analgesia. To assess the clinical analgesic efficacy, pharmacodynamic studies are essential. The experimental model employed in this study is a validated approach for determining nociception in chicken embryos, and has identified MAP as a cardiovascular and wide beak opening as a behavioral, sensitive nociceptive parameter in chicken embryos [9,10]. Regarding the MAP, our data confirm this finding, as all NaCl-treated groups responded to the Pinch with a significant increase in MAP, as a nociceptive reaction to a noxious stimulus, ranging from 10.96% to 14.12%. Administration of fentanyl or buprenorphine into the air chamber did not lead to a significant reduction in MAP increase. It must be noted that, particularly in the NaCl groups, a higher variance occurred than estimated in the prior power analysis. As a result, an actual effect may not have been statistically recorded. This would be supported by the observed medium to large effect sizes, which may suggest a difference of clinical importance. As pain is a highly individual experience [39,40], not all chicken embryos showed the same distinct reaction to a noxious stimulus. In addition, despite a high degree of standardization, other factors may have played a role in the different responses to the Pinch. The sensitive organism could have been further stressed by the invasive methodology, light and in some cases bleeding caused by the preparation of the CAM.

**Fig 6:**
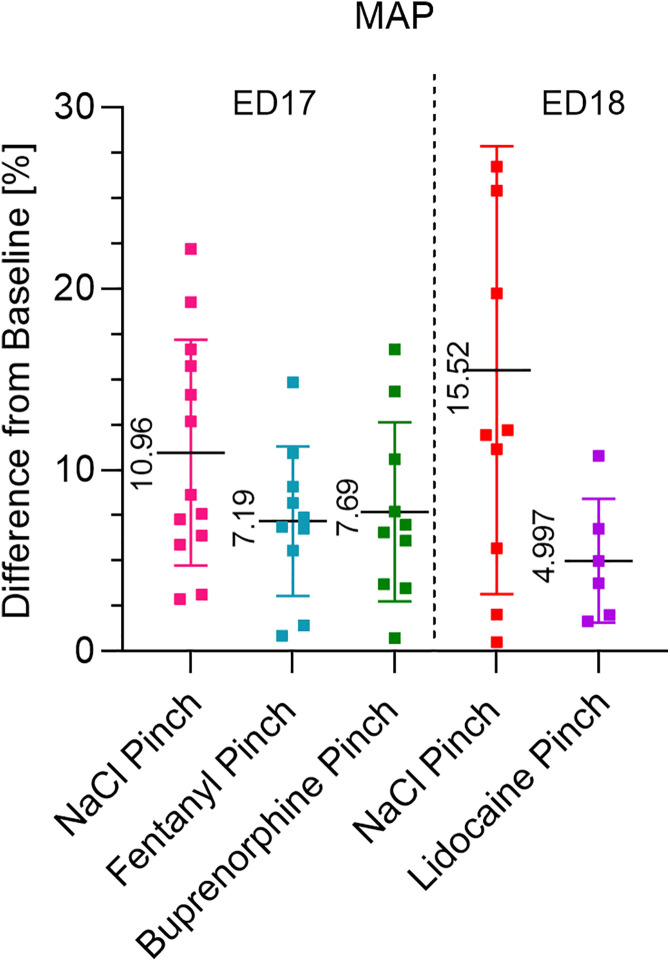
A comparison: Percent Change in MAP after Pinch with fentanyl/buprenorphine (ED17) and lidocaine (ED18). ED17 embryos that received a Pinch 15 min after application of NaCl, fentanyl and buprenorphine compared to ED18 embryos being pinched after injection of NaCl and lidocaine. The percent change from BL in MAP after Pinch is presented. Values are shown as the mean ± SD. Infiltration anesthesia by injection of lidocaine at the base of the beak led to a significant reduction of the MAP increase after Pinch [9].

In veterinary medicine, rescue analgesia is administered when an increase in HR or BP of approximately 20% is observed [41]. We were not able to provoke these strong reactions in MAP or HR in chicken embryos. Although the development of the heart of chicken embryos is already quite advanced at ED17, the maturation of the heart is only completed after hatching [42]. Since the myocardium of newborn mammals is not yet so flexible, an increase in cardiac output cannot be achieved by increasing the contractility of the heart, but only by increasing the heart rate [43]. The mechanisms for BP regulation are therefore probably not yet fully developed due to the incompletely mature cardiovascular system and regulation of the chicken embryos. This is supported by our previous study, where we observed an increase in the MAP reaction with each additional day of incubation [9]. In a study in which a cold pressor test was performed in adult humans following i.v. administration of fentanyl at a dose of 1 µg/kg the increase in systolic BP could be reduced from 13 mmHg to 7 mmHg [44]. With this dosage, a reduction of approximately 50% was achieved. However, this test was carried out on fully developed individuals, making direct comparison with embryonic models difficult. Infiltration anesthesia with lidocaine significantly reduced MAP increase in ED18 embryos by approximately 67% [9] (Fig 6). This degree of reduction may serve as a useful reference point, as lidocaine representing local anesthetics met the required pharmacological profile and provided rapid local pain relief [45]. However, it must be taken into account that these embryos were one day older and the natural MAP reaction to the Pinch was slightly higher [9].

Opiate receptors are detectable in brain and body tissue in chicken embryos as early as ED4 [46]. A developmental change in the µ-opioid receptors was observed from early to later embryonic stages [47]. This was interpreted as a maturation to the adult pattern, which seems to be completed on ED18 [47].

The presence of µ-, κ- and δ-opioid receptor mRNA in cardiomyocytes of ED15 chicken embryos was confirmed by RT-PCR [48]. µ- and κ-opioid receptors were also detected in numerous brain regions in one-day-old chicks [49]. The central nervous system has been identified as the main localization for the expression of the opioid system in adult chicken [50]. However, the mere presence of the receptors does not confirm their functionality. Successful inhibition of Substance P release by µ-, κ- and δ-opioid receptor agonists has demonstrated the functionality of opioid receptors in ED10 - ED12 chicken embryo tissue [51]. This finding makes the use of the opioid analgesics fentanyl and buprenorphine appear promising from this developmental time point onwards.

Despite evidence of the presence of opioid receptors in chicken embryos [48], their absolute number may be too low for the drugs to be fully effective. It may also be possible that the opioid receptors are not yet mature enough for optimal binding capacity [47]. Hoppes et al. suggest that the analgesic effect of fentanyl at higher doses of 0.2 mg/kg in White Cockatoos may be explained by receptor crossover, activating other opioid receptors (non µ) [36]. This phenomenon has also been suspected for buprenorphine [52]. Thus, increasing the opioid doses in chicken embryos may enhance the effect on MAP response. However, buprenorphine is known for a ceiling effect, which means that no greater effect can be achieved above a certain dose [53].

Due to the opioids´ ability to induce bradycardia and sedation [27], they were also investigated with regard to their side effect potential. Neither fentanyl nor buprenorphine showed cardiovascular or sedative side effects in the doses used. The data suggest that the applied drugs were well tolerated at the tested dosages.

Wide beak opening, as a nocifensive behavioral reaction of chicken embryos to a noxious stimulus, is reduced by lidocaine infiltration [10]. The number of NaCl-treated embryos at 15 min showing wide beak openings was significantly more frequent after Pinch than after Touch and after application of fentanyl (15 min). This strengthens a possible anti-nociceptive effect of fentanyl at this time, which also corresponds to the measured C_max_ at 15 min. The reason why no reduction was observed at other time points and when buprenorphine was used could be due to differences in the onset of analgesia and the potency between fentanyl and buprenorphine. While the effect of the more potent fentanyl starts 3–5 min after injection and lasts for 20–30 min, the effect of buprenorphine starts after 30–60 min and lasts for 8–10 h [22,54]. In this study embryo movements were counted by direct observation during the experiment and not afterwards on the basis of video recordings [10]. Despite all efforts, there was a risk that movements were missed. The variability in body movements could also be explained by the random occurrence of spontaneous uncoordinated, and even coordinated movements described as preparatory for hatching [55,56]. Further, the preparation of the egg for the insertion of the measuring instruments may have caused individual stress, which may have influenced the movements of the embryo.

A limitation of this study is that experiments were only conducted on ED17 embryos and only in the Leghorn White breed. A generalization of the results is therefore not possible. As there are large interspecies and breed-specific differences in analgesia in birds, further studies must be performed in different breeds, as a simple transmission of the data might be difficult [57]. These future studies should also include higher doses of the opioids. In addition, earlier and later stages of development, should be included to determine plasma concentrations, analgesic efficacy and tolerability.

## 5. Conclusions

Pharmacokinetic profiles were analyzed for ED17 chicken embryos for fentanyl (0.2 mg/kg) and buprenorphine (0.5 mg/kg). Maximum plasma levels were reached 15 min following administration of both substances. The maximum concentrations determined exceeded plasma concentrations considered therapeutically effective in humans. Plasma levels previously measured in birds were achieved and analgesic efficacy was tested, which was neglected in many previous studies. Even though statistically significant reductions in MAP were not achieved with either opioid analgesic, the observed moderate to large effect sizes, together with the significant reduction in wide beak opening—particularly with fentanyl—may indicate a potential anti-nociceptive effect.

Further studies with higher doses and at different developmental days are required to investigate the clinical analgesic efficacy in the chicken embryo.

## Supporting information

S1 Table Overview of egg weight, embryo weight and sex distribution – pharmacokinetics. (PDF)

S2 Table Overview of egg weight, embryo weight and sex distribution – pharmacodynamics. (PDF)

S3 Table Overview of effect sizes – pharmacodynamics. (PDF)

S1 Fig Fentanyl plasma concentration (ng/ml) at 5, 15, 60, and 120 min after application via air chamber (left) or chorioallantoic membrane (CAM; right); n = 2–3 per time point. (PDF)

S2 Fig Percent change in HR after mechanical stimuli with fentanyl and buprenorphine. (PDF)
