# Supplementary material for: Pharmacokinetics and analgesic efficacy of fentanyl and buprenorphine in chicken embryos
Source: PLoS One. 2026 Jan 8;21(1):e0340576. doi: 10.1371/journal.pone.0340576 (PMC12782372; doi:10.1371/journal.pone.0340576)
Supplement: S1 Table — (PDF) [file pone.0340576.s001.pdf]

S1 Table: Overview of Egg Weight, Embryo Weight and Sex Distribution – Pharmacokinetics.

	Fentanyl						Buprenorphine			
	5 min	15 min	30 min	45 min	60 min	120 min	15 min	30 min	60 min	120 min
Egg weight (g)	42.07 ± 3.89	41.93 ± 3.53	43.55 ± 3.99	46.90 ± 0.92	42.53 ± 5.97	41.70 ± 5.18	49.80 ± 3.27	51.31 ± 2.95	51.76 ± 3.04	53.77 ± 1.96
Embryo weight (g)	15.09 ± 1.09	14.62 ± 2.11	15.90 ± 1.36	17.47 ± 0.76	15.80 ± 1.65	14.33 ± 2.58	15.07 ± 4.03	14.57 ± 4.57	14.87 ± 2.35	14.87 ± 3.27
Sex (m/f)	6/4	6/5	3/5	1/2	3/1	3/1	3/4	4/3	2/5	2/5

Egg weight (g), embryo weight (g) and sex (male/female) (m/f) of chicken embryos of the pharmacokinetic test groups at ED17. Values are shown as the mean ± standard deviation (SD) for egg and embryo weight. The absolute numbers of the respective sexes are indicated. Egg and embryo weight did not differ significantly within either the fentanyl or buprenorphine group, as determined by ordinary one way ANOVA or Kruskal–Wallis tests.
