# Supplementary material for: Pharmacokinetics and analgesic efficacy of fentanyl and buprenorphine in chicken embryos
Source: PLoS One. 2026 Jan 8;21(1):e0340576. doi: 10.1371/journal.pone.0340576 (PMC12782372; doi:10.1371/journal.pone.0340576)
Supplement: S2 Table — (PDF) [file pone.0340576.s002.pdf]

S2 Table: Overview of Egg Weight, Embryo Weight and Sex Distribution - Pharmacodynamics.

	15 min			30 min			60 min		
	NaCl15	Buprenorphine15	Fentanyl 15	NaCl30	Buprenorphine30	Fentanyl 30	NaCl60	Buprenorphine60	Fentanyl 60
Egg weight (g)	48.29[a] ± 4.56	46.59[a] ± 2.83	45.04 ± 5.41	46.88[a] ± 2.88	47.04[a] ± 1.74	45.49 ± 2.20	43.88 ± 3.06	45.69 ± 2.94	41.97[b] ± 2.04
Embryo weight (g)	15.12 ± 2.64	15.93 ± 1.78	15.17 ± 2.12	15.13 ± 2.29	14.97 ± 2.62	14.89 ± 1.62	15.53 ± 1.41	15.74 ± 2.72	14.63 ± 1.02
Sex (m/f)	5/8	6/4	2/8	4/9	5/5	5/5	6/7	3/7	6/4

Egg weight (g), embryo weight (g) and sex (male/female) (m/f) of chicken embryos of the different test groups at ED17. Values are shown as the mean ± SD for egg and embryo weight. The absolute numbers of the respective sexes are indicated. Egg weight was analyzed using the Kruskal-Wallis test, for embryo weight an ordinary one-way ANOVA was performed. Data that showed significance were further tested using the post-hoc Tukey test (for ANOVA) and the post-hoc Dunn test (for Kruskal-Wallis). The data of all groups for egg and embryo weight were compared with each other. Significant differences in egg weight ($p < 0.05$), indicated by different superscripts in a row, were observed between Fentanyl60 and NaCl15 ($p = 0.0016$), Buprenorphine15 ($p = 0.0293$), NaCl30 ($p = 0.0222$) as well as Buprenorphine30 ($p = 0.0094$).
