# Supplementary material for: Pharmacokinetics and analgesic efficacy of fentanyl and buprenorphine in chicken embryos
Source: PLoS One. 2026 Jan 8;21(1):e0340576. doi: 10.1371/journal.pone.0340576 (PMC12782372; doi:10.1371/journal.pone.0340576)
Supplement: S3 Table — (PDF) [file pone.0340576.s003.pdf]

S5 Table: Overview of Effect Sizes – Pharmacodynamics.

Calculated Hedges' g effect sizes for percent change in MAP and HR at 15, 30 and 60 min .

a

MAP Hedges' g			
	15 min	30 min	60 min
NaCl Touch vs. Fentanyl Touch	0.58	0.47	0.55
NaCl Touch vs. NaCl Pinch	1.49	1.43	1.80
NaCl Touch vs. Fentanyl Pinch	1.07	1.89	0.94
Fentanyl Touch vs. NaCl Pinch	1.79	1.46	1.94
Fentanyl Touch vs. Fentanyl Pinch	1.52	2.06	1.26
NaCl Pinch vs. Fentanyl Pinch	0.69	0.55	1.09

b

MAP Hedges' g			
	15 min	30 min	60 min
NaCl Touch vs. Buprenorphine Touch	0.77	0.30	1.32
NaCl Touch vs. NaCl Pinch	1.49	1.43	1.80
NaCl Touch vs. Buprenorphine Pinch	1.06	1.14	0.94
Buprenorphine Touch vs. NaCl Pinch	1.85	1.42	2.17
Buprenorphine Touch vs. Buprenorphine Pinch	1.57	1.18	1.66
NaCl Pinch vs. Buprenorphine Pinch	0.57	0.63	1.04

c

HR Hedges' g			
	15 min	30 min	60 min
NaCl Touch vs. Fentanyl Touch	0.14	0.43	0.43
NaCl Touch vs. NaCl Pinch	1.45	0.89	1.21
NaCl Touch vs. Fentanyl Pinch	0.67	0.97	1.14
Fentanyl Touch vs. NaCl Pinch	1.73	1.09	0.77
Fentanyl Touch vs. Fentanyl Pinch	0.91	1.22	0.70
NaCl Pinch vs. Fentanyl Pinch	0.76	0.02	0.05

d

HR Hedges' g			
	15 min	30 min	60 min
NaCl Touch vs. Buprenorphine Touch	0.19	0.33	0.03
NaCl Touch vs. NaCl Pinch	1.45	0.89	1.21
NaCl Touch vs. Buprenorphine Pinch	1.12	1.38	1.85
Buprenorphine Touch vs. NaCl Pinch	1.68	0.72	1.13
Buprenorphine Touch vs. Buprenorphine Pinch	1.29	1.21	1.68
NaCl Pinch vs. Buprenorphine Pinch	0.13	0.38	0.05
