# Supplementary material for: Pharmacokinetics and analgesic efficacy of fentanyl and buprenorphine in chicken embryos
Source: PLoS One. 2026 Jan 8;21(1):e0340576. doi: 10.1371/journal.pone.0340576 (PMC12782372; doi:10.1371/journal.pone.0340576)

S3 Fig: Fentanyl plasma concentration (ng/ml) at 5, 15, 60, and 120 min after application of 0.2 mg/kg egg weight via air chamber (left) or chorioallantoic membrane (CAM; right); n = 2-3 per time point.

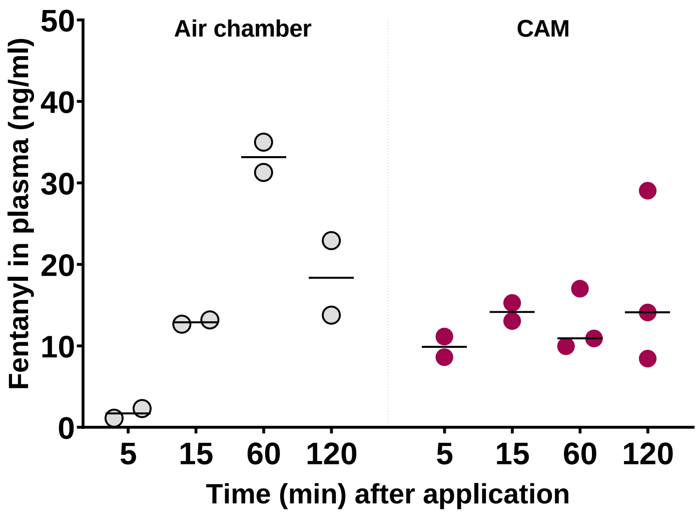

Supplement: S1 Fig — (PDF) [file pone.0340576.s004.pdf]
