# Supplementary material for: Pharmacokinetics and analgesic efficacy of fentanyl and buprenorphine in chicken embryos
Source: PLoS One. 2026 Jan 8;21(1):e0340576. doi: 10.1371/journal.pone.0340576 (PMC12782372; doi:10.1371/journal.pone.0340576)
Supplement: S2 Fig — (PDF) [file pone.0340576.s005.pdf]

S4 Fig: Percent Change in HR after Mechanical Stimuli with Fentanyl and Buprenorphine.

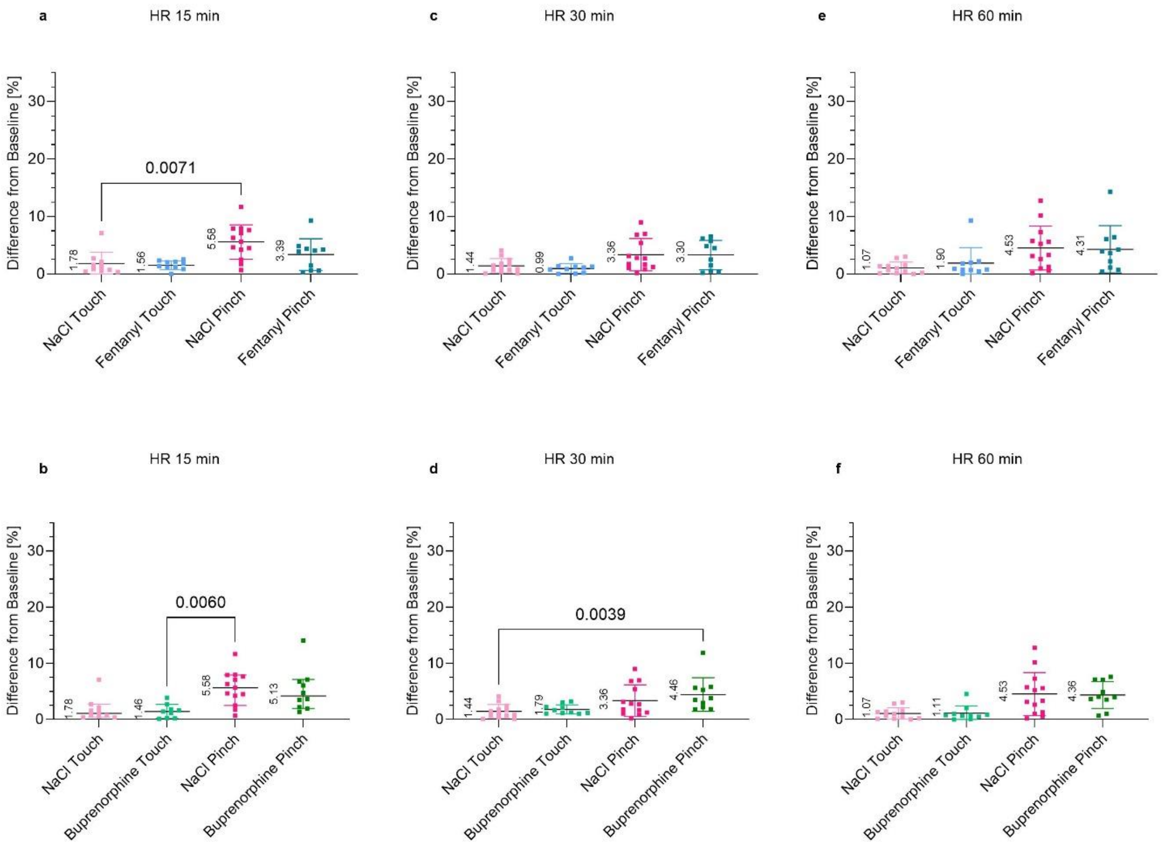

ED17 embryos received a mechanical stimulus (Pinch/Touch) at different time points (15, 30, 60 min) after administration of NaCl (a-f), fentanyl (a, c, e) or buprenorphine (b, d, f). **(a-f)** Percent change from BL in MAP after Pinch and Touch. Values are shown as the mean ± SD. Normally distributed data were analyzed by ordinary one-way ANOVA (c), non-normally distributed data by a Kruskal-Wallis test (a, b, d, e, f). A p-value of < 0.01 was considered statistically significant.
